# Risk analysis of COVID-19 hospitalization and critical care by race and region in the United States: a cohort study

**DOI:** 10.1186/s12889-023-16401-4

**Published:** 2023-08-04

**Authors:** Mitsuki Jimbo, Sakae Saito, Takayuki Uematsu, Hideaki Hanaki, Katsuya Otori, Kiyoshi Shibuya, Wataru Ando

**Affiliations:** 1 Department of Clinical Pharmacy, Center for Clinical Pharmacy and Sciences, Kitasato University School of Pharmacy, Minato-Ku, Tokyo, Japan; 2 Department of Pharmacy, Kitasato University Medical Center, Kitamoto City, Saitama, Japan; 3 Biomedical Laboratory, Division of Biomedical Research, Kitasato University Medical Center, Kitamoto City, Saitama, Japan; 4 Infection Control Research Center, Ōmura Satoshi Memorial Institute, Kitasato University, Minato-Ku, Tokyo, Japan; 5 Laboratory of Pharmacy Practice and Science 1, Division of Clinical Pharmacy, Research and Education Center for Clinical Pharmacy, Kitasato University School of Pharmacy, Sagamihara, Japan

**Keywords:** COVID-19, Hospitalization, Critical care, Cox proportional hazards model, United States

## Abstract

**Background:**

This study aimed to identify the current risk factors for coronavirus disease 2019 severity and examine its association with medication use.

**Methods:**

We used data from a large United States electronic health record database to conduct an anonymized cohort study of 171,491 patients with coronavirus disease 2019. The study was conducted from January 1, 2020, to August 27, 2021. Data on age, race, sex, history of diseases, and history of medication prescriptions were analyzed using the Cox proportional hazards model analysis to calculate hazard ratios for hospitalization and severe risk.

**Results:**

Factors that increased the risk of hospitalization and critical care were age ≥ 65 years, male sex, type 2 diabetes, hypertension, interstitial pneumonia, and cardiovascular disease. In particular, age ≥ 65 years significantly increased the risk of hospitalization (hazard ratio, 2.81 [95% confidence interval, 2.58–3.07]; *P* < 0.001) and critical care (hazard ratio, 3.45 [2.88–4.14]; *P* < 0.001). In contrast, patients with hyperlipidemia had a reduced risk. However, patients with hyperlipidemia who were not taking statins had a significantly increased risk of hospitalization (hazard ratio, 1.24 [1.16–1.34]; *P* < 0.001). Sodium-glucose cotransporter-2 inhibitors, angiotensin-converting enzyme inhibitors, angiotensin II receptor blockers, glucocorticoids, and statins significantly reduced the risk of hospitalization and critical care. The risk of hospitalization and critical care increased in patients of all ethnicities with type 2 diabetes. The factors that significantly increased the risk of hospitalization in all regions were older age, hypertension, chronic obstructive pulmonary disease, and cardiovascular disease.

**Conclusion:**

This study identified factors that increase or reduce the risk of severe coronavirus disease. The provision of appropriate drug treatment and modification of lifestyle-related risk factors may reduce coronavirus disease severity.

## Introduction

Studies on the risk factors for severe coronavirus disease 2019 (COVID-19) have been conducted worldwide in patients with varying demographic characteristics. Older age, male sex, obesity, and certain diseases such as hypertension and diabetes have been reported as risk factors for severe disease [1–3]. Several studies indicate that there are differences in the risk of severe disease among races and regions [4–6]. According to the 2020 United States Census, 57.8% of the population is non-Hispanic White, 18.7% is Hispanic, 12.1% is non-Hispanic Black, and 6% is non-Hispanic Asian. Obesity prevalence is 41%, diabetes is 10%, and hypertension is 47% [7]. Therefore, many Americans have high-risk factors for COVID-19.

Several studies have shown that drug therapy for the underlying disease is not associated with a substantial increase in the risk of COVID-19 severity, suggesting that metformin, sodium-glucose cotransporter-2 (SGLT2) inhibitors, and beta blockers may improve the conversion [8, 9]. There are reports that statins and angiotensin-converting enzyme (ACE) inhibitors/angiotensin II receptor blockers (ARBs) have reduced the risk of COVID-19 severity [10, 11]. Patients with risk factors for COVID-19 severity could be at a reduced risk with these agents; however, the relationship between the risk of COVID-19 severity and race, region, underlying disease, and medication has not been adequately investigated. Many previous studies have used data from the early stages of the pandemic, which may not reflect the most recent situation. Therefore, this study aimed to use information available from a large United States electronic health record (EHR) database to identify current risk factors for COVID-19 severity, and to examine the association between medication and COVID-19 severity.

## Materials and methods

### Study design and date source

This study used medical and pharmacy claims data and electronic medical records from the Healthjump database (Healthjump Inc., Philadelphia, PA, USA) provided by the COVID-19 Research Database Consortium. All personal information in this database is anonymized. The Healthjump configuration tables consist of demographic (patient background), diagnosis (diagnosis name), medication, and procedure (treatment) data variables. Data were extracted by SQL using Snowflake (Snowflake Inc., San Mateo, CA, USA).

### Data analysis

From January 1, 2020, to August 27, 2021, in the United States, 171,491 patients who had (1) a diagnosis of COVID-19 (International Statistical Classification of Diseases and Related Health Problems, Tenth Revision; ICD-10: U17.1) and (2) were aged 20 years or older were included in this study. Patients were excluded if they (1) were younger than 20 years of age; (2) had missing race and sex; (3) had a body mass index (BMI) ≤ 15 kg/m^2^ or BMI > 50 kg/m^2^; or (4) had suspected COVID-19, but no confirmed diagnosis. Data obtained from the EHR included date of diagnosis, race (White, Black, Asian, other, or unknown), ethnicity (Hispanic, non-Hispanic, or unknown), region (Northeast, Midwest, South, or West), hospitalization and critical care status, age, sex, and BMI (value on the date closest to the date of diagnosis in the past 6 months). In addition, data on history of diseases that are considered to be significant risk factors for COVID-19 severity (type 2 diabetes [ICD-10: E11.65, E11.8, E11.9], hypertension [ICD-10: I10], hyperlipidemia [ICD-10: E78], obesity [BMI > 30 kg/m^2^, as defined by the Centers for Disease Control and Prevention], asthma [ICD-10: J45], chronic obstructive pulmonary disease [COPD] [ICD-10: J44], interstitial pneumonia [ICD-10: J84], cardiovascular disease [angina pectoris, myocardial infarction, heart failure, and other ischemic heart disease] [ICD-10: I20, I21, I22, I23, I24, I25, I50]) and prescription history (whether or not prescribed in the year prior to diagnosis) of drugs to treat these conditions were included. This analysis was conducted based on the methodology used in previous studies [12, 13].

Each state was classified into four regions according to the United States Census Bureau regional classifications (https://www.census.gov). The District of Columbia (Washington, DC) is not part of any state, but is considered Southern; therefore, it was classified as Southern in this study.

If more than one value was obtained for each dataset, the value obtained at the time closest to the diagnosis of COVID-19 was considered. To evaluate hospitalization rates and critical care rates, the Current Procedural Terminology (CPT) fourth edition (CPT-4) codes and Healthcare Common Procedure Coding System (HCPCS) codes used for reimbursement in the United States were used as references. Patients with a code of hospitalization (99,221–99,223 in CPT4 and HCPCS) were defined as hospitalized, those with a code of critical care (99,291–99,293 in CPT4 and HCPCS) were defined as critical care, and patients without either of the above codes were defined as having no admission. The oldest code was used in place of the CPT4 and HCPCS codes, for diagnosis in cases where the same code or codes before and after diagnosis were consecutive or overlapping.

We used these codes for analysis if the patient had these codes for less than 30 days after being diagnosed with COVID-19. Sub-analyses were conducted for each region in the United States and race, with the objective of determining characteristics by race and region.

### Statistical analysis

Statistical analyses were performed using STATA 16.0 (Stata Corp. LCC., Lakeway, TX, USA). Risk factors for hospitalization and critical care were analyzed using Cox proportional hazards model analysis, and hazard ratios (HRs) were calculated. Univariate analysis was performed with the extracted codes (presence or absence) set as the independent variables; multivariate analysis was performed for factors with *P* ≤ 0.2. The significance level for multivariate analysis was set at *P* < 0.05. The same analysis was performed for hospitalization and critical care by race and region.

## Results

### Clinical characteristics

The clinical characteristics of the patients, number of patients with each disease, and number of patients using each drug for each disease are shown in Table 1. Among 171,492 COVID-19 patients, 69,887 (40%) were men, 95,739 (56%) were women, and 56 (< 0.1%) were other. The mean age (± standard deviation) was 51.0 ± 17.6 years and the mean BMI (± standard deviation) was 31.2 ± 9.4 kg/m^2^. The number of hospitalized patients and those who received critical care services were 5,812 (3.4%) and 1,704 (1.0%), respectively. The number of patients by race was White, 92,184 (53.8%); Black, 21,052 (12.3%); and Asian, 1,440 (0.8%). The number of patients per region was 19,909 in the Northeast (11.6%), 10,612 in the Midwest (6.2%), 100,574 in the South (58.6%), and 40,299 in the West (23.5%).

**Table 1:** Characteristics of patients with COVID-19 in hospitalization and critical care

	Total	Hospitalization	Critical care
Number of patients, n (%)	171,491	5812 (3.4)	1704 (1.0)
Age, mean (SD), years	51.0 (17.6)	64.9 (16.6)	64.5 (17.6)
Sex (male/female)	73,162/98609	3107/2702	1035/669
BMI mean (SD), kg/m^2^	32.6 (9.4)	32.5 (10.6)	32.8 (9.4)
BMI ≥ 30 kg/m^2^, n (%)	72,387	1871 (2.6)	503 (0.7)
Area of residence			
Northeast, n (%)	19,909	606 (3.0)	33 (0.2)
Midwest, n (%)	10,612	151 (1.4)	3 (0)
South, n (%)	100,574	3644 (3.6)	1215 (1.2)
West, n (%)	40,299	1411 (3.5)	453 (1.1)
unknown, n (%)	97	0	0
Race and Ethnicity			
White, n (%)	92,184	2282 (2.5)	437 (0.5)
Black, n (%)	21,052	963 (4.6)	330 (1.6)
Asian, n (%)	1440	57 (4.0)	13 (0.9)
others, n (%)	3336	17 (0.5)	6 (0.2)
Hispanic, n (%)	39,553	704 (1.8)	68 (0.2)
unknown, n (%)	13,926	1789 (12.8)	850 (6.1)
T2D and Medications			
T2D, n (%)	31,191	1839 (5.9)	540 (1.7)
Alpha-glucosidase inhibitor, n (%)	10	1 (10.0)	0
DPP4 inhibitor, n (%)	59	0	0
Incretin mimetic, n (%)	3849	96 (2.5)	29 (0.8)
Insulin, n (%)	237	7 (3.0)	2 (0.8)
Meglitinide, n (%)	32	4 (12.5)	0
Metformin, n (%)	4408	106 (2.4)	22 (0.5)
SGLT2 inhibitor, n (%)	3213	81 (2.5)	15 (0.5)
Sulfonylurea, n (%)	2457	54 (2.2)	11 (0.4)
Thiazolidinedione, n (%)	654	19 (2.9)	5 (0.8)
Hypertension and Medications			
Hypertension, n (%)	65,360	3143 (4.8)	796 (1.2)
Calcium channel blockers, n (%)	5726	183 (3.2)	27 (0.5)
ACE inhibitors, n (%)	6571	136 (2.1)	11 (0.2)
ARBs, n (%)	5442	114 (2.1)	21 (0.4)
Respiratory Diseases and Medications			
Asthma, n (%)	16,528	432 (2.6)	119 (0.7)
COPD, n (%)	7334	623 (8.5)	160 (2.3)
Interstitial pneumonia, n (%)	1063	116 (10.9)	24 (2.3)
Beta^2^ stimulants, n (%)	10,077	200 (2.0)	24 (0.2)
Theophylline, n (%)	21	0	0
Inhaled glucocorticoids, n (%)	21,839	347 (1.6)	59 (0.3)
Mineralocorticoid, n (%)	48	3 (6.3)	0
Inhaled anticholinergics, n (%)	3115	142 (4.6)	24 (0.8)
Beta stimulator + steroids combinations, n (%)	6829	225 (3.3)	46 (0.7)
Hyperlipidemia and Medications			
Hyperlipidemia, n (%)	66,628	2029 (3.0)	472 (0.7)
Bile acid adsorbent, n (%)	431	11 (2.6)	2 (0.5)
Ezetimibe, n (%)	837	18 (2.2)	4 (0.5)
Fibrates, n (%)	832	15 (1.8)	8 (1.0)
Other lipid-lowering drugs, n (%)	784	16 (2.0)	4 (0.5)
PCSK9 inhibitors, n (%)	197	7 (3.6)	1 (0.5)
Statins, n (%)	12,517	314 (2.5)	45 (0.4)
Combination drugs containing statin, n (%)	77	1 (1.3)	0
Cardiovascular disease and Medications			
Cardiovascular disease, n (%)	15,783	1419 (9.0)	454 (2.9)
Antiplatelet agents, n (%)	3250	118 (3.6)	16 (0.5)
PAR-1 inhibitors, n (%)	1	0	0
Beta blockers, n (%)	3927	154 (3.9)	32 (0.8)
Beta blockers + Thiazolidinedione, n (%)	134	8 (6.0)	0

*ACE* Angiotensin-converting enzyme, *ARB* Angiotensin II receptor blockers, *BMI* Body mass index, *COPD* Chronic obstructive pulmonary disease, *COVID-19* Coronavirus disease 2019, *DPP-4* Dipeptidyl peptidase 4, *PAR-1* Protease activated receptor-1, *PCSK9* Proprotein convertase subtilisin/kexin type 9, *SD* Standard deviation, *SGLT2* Sodium-glucose cotransporter-2, *T2D* Type 2 diabetes

### Hospitalization and critical care risks

**Fig. 1:**
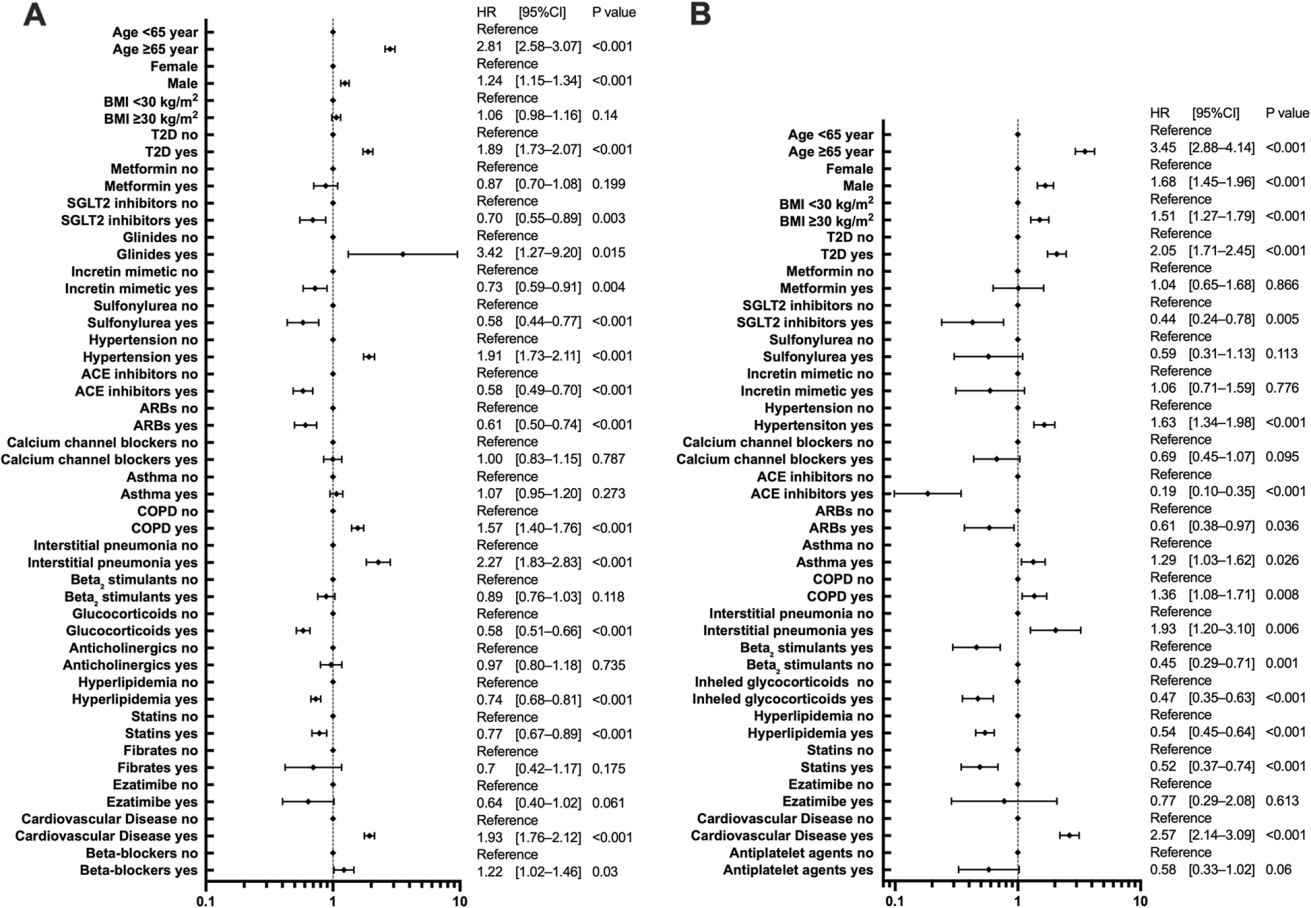
Risk analysis of hospitalization and critical-care factors for COVID-19 using the Cox proportional hazards model. The forest plot shows the HRs (diamonds) and 95% CIs (horizontal bars) for hospitalization risk (**A**) and critical care (**B**). ACE, angiotensin-converting enzyme; ARB, angiotensin receptor blocker; BMI, body mass index; CI, confidence interval; COPD, chronic obstructive pulmonary disease; COVID-19, coronavirus disease 2019; HR, hazard ratio; SGLT2, sodium-glucose cotransporter-2; T2D, type 2 diabetes

The results of the multivariate analysis based on the univariate results for the risks of hospitalization and critical care are shown in Fig. 1. The analysis showed that the factors that significantly increased the risk of both hospitalization and critical care were age ≥ 65 years, male sex, type 2 diabetes, hypertension, interstitial pneumonia, and cardiovascular diseases. In particular, age ≥ 65 years significantly increased the risk of hospitalization (HR 2.81, 95% confidence interval [CI] [2.58–3.07], *P* < 0.001) and critical care (HR 3.45 [2.88–4.14], *P* < 0.001). In contrast, SGLT2 inhibitors, ACE inhibitors, ARBs, glucocorticoids, and statins significantly reduced the risk of hospitalization and critical care. In our analysis, hyperlipidemia also significantly reduced the risk of hospitalization and critical care (HR 0.74 [0.68–0.81], *P* < 0.001/HR 0.54 [0.45–0.64], *P* < 0.001).

### Sub-analysis of hyperlipidemia

As the risk was reduced in patients with hyperlipidemia, a detailed analysis was performed.

**Fig. 2:**
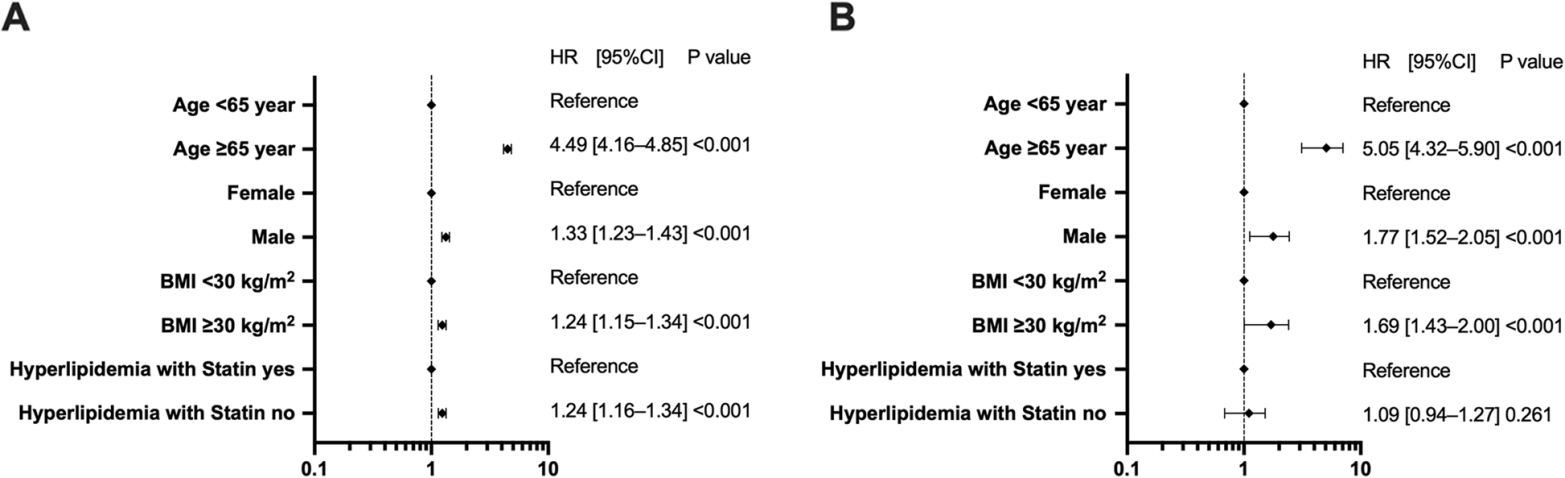
Sub-analysis of hospitalization and critical-care factors in COVID-19 patients with hyperlipidemia. The forest plot shows the HRs (diamonds) and 95% CIs (horizontal bars) for hospitalization risk (**A**) and critical care (**B**). BMI, body mass index; CI, confidence interval; COVID-19, coronavirus disease 2019; HR, hazard ratio

Of the patients with hyperlipidemia, 12,517 were prescribed statins and 54,111 were not. The results for the risk of hospitalization and critical care are shown in Fig. 2. The analysis showed an HR of 1.24 [95% CI: 1.15–1.34] (*P* < 0.001) for hospitalization in patients not prescribed statins. Patients with hyperlipidemia who were not taking statins had a significantly increased risk of hospitalization (Fig. 2a). In contrast, the HR for critical care was 1.09 [95% CI: 0.94–1.27] *(P* = 0.261) (Fig. 2b). No significant differences were found, although there was a trend toward increased risk.

### Differences in risk of hospitalization and critical care among different races

**Fig. 3:**
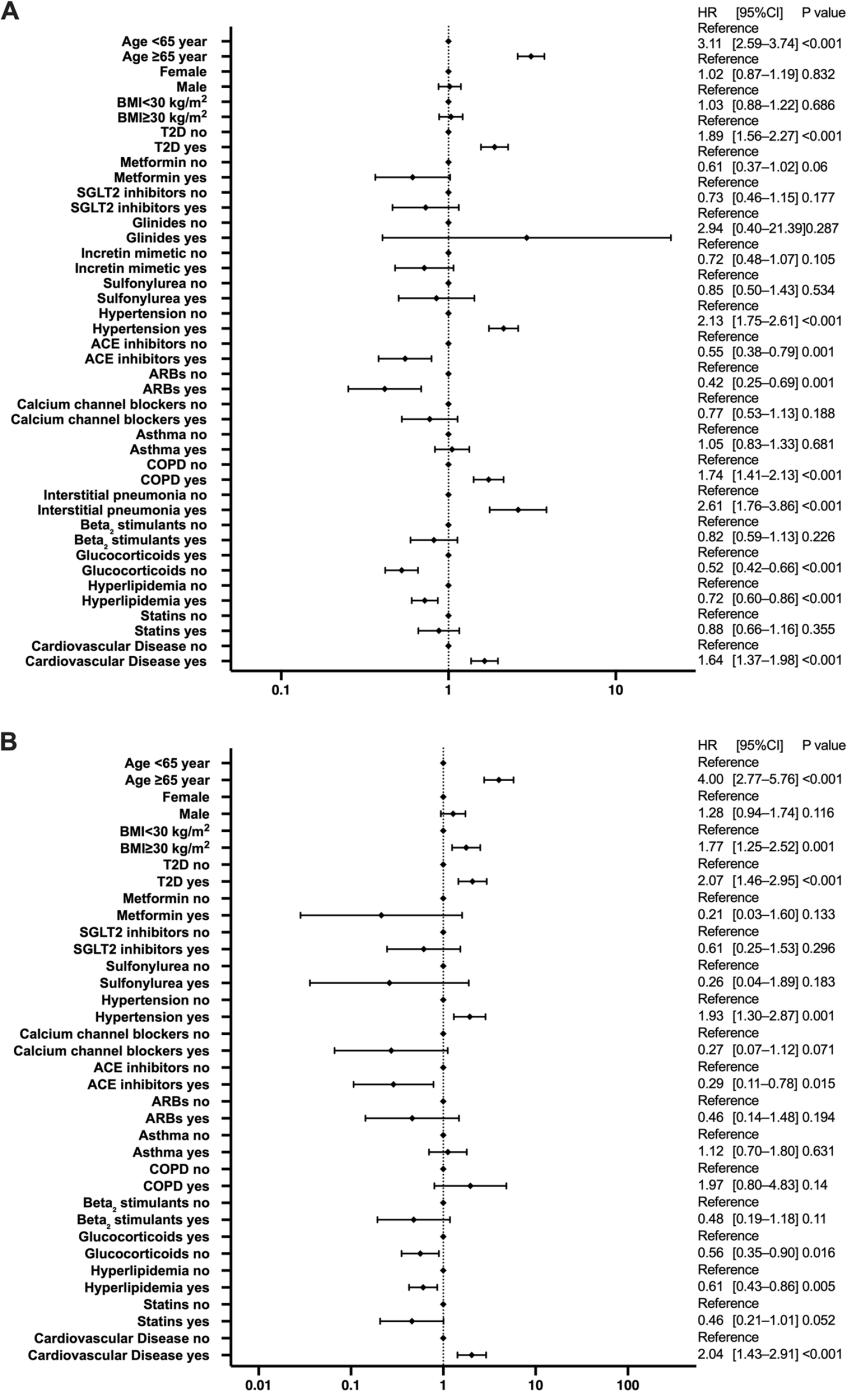
Analysis of hospitalization and critical-care factors in non-Hispanic White COVID-19 patients. The forest plot shows the HRs (diamonds) and 95% CIs (horizontal bars) for hospitalization risk (**A**) and critical care (**B**). ACE, angiotensin-converting enzyme; ARB, angiotensin receptor blocker; BMI, body mass index; CI, confidence interval; COPD, chronic obstructive pulmonary disease; COVID-19, coronavirus disease 2019; HR, hazard ratio; SGLT2, sodium-glucose cotransporter-2; T2D, type 2 diabetes

**Fig. 4:**
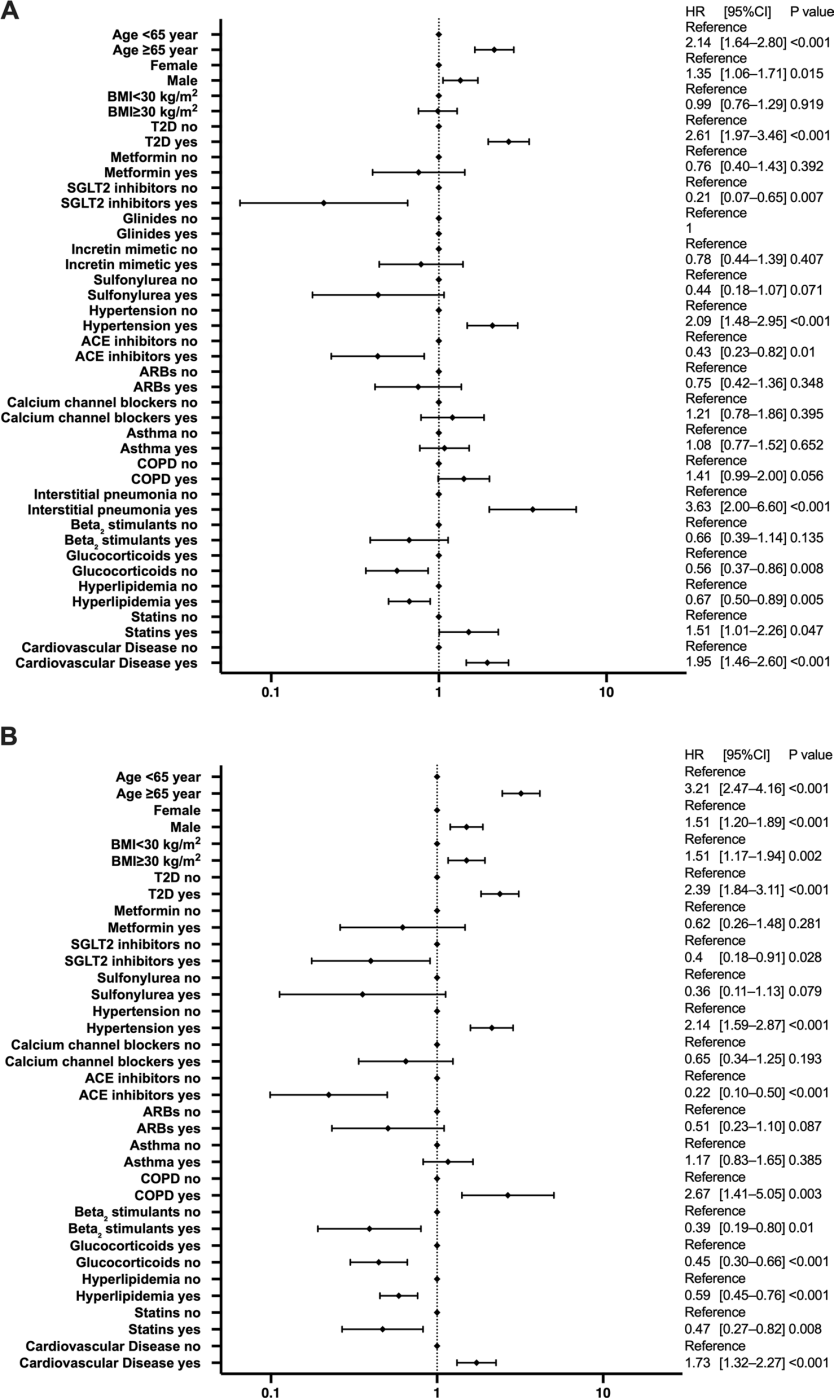
Analysis of hospitalization and critical-care factors in non-Hispanic Black COVID-19 patients. The forest plot shows the HRs (diamonds) and 95% CIs (horizontal bars) for hospitalization risk (**A**) and critical care (**B**). ACE, angiotensin-converting enzyme; ARB, angiotensin receptor blocker; BMI, body mass index; CI, confidence interval; COPD, chronic obstructive pulmonary disease; COVID-19, coronavirus disease 2019; HR, hazard ratio; SGLT2, sodium-glucose cotransporter-2; T2D, type 2 diabetes

**Fig. 5:**
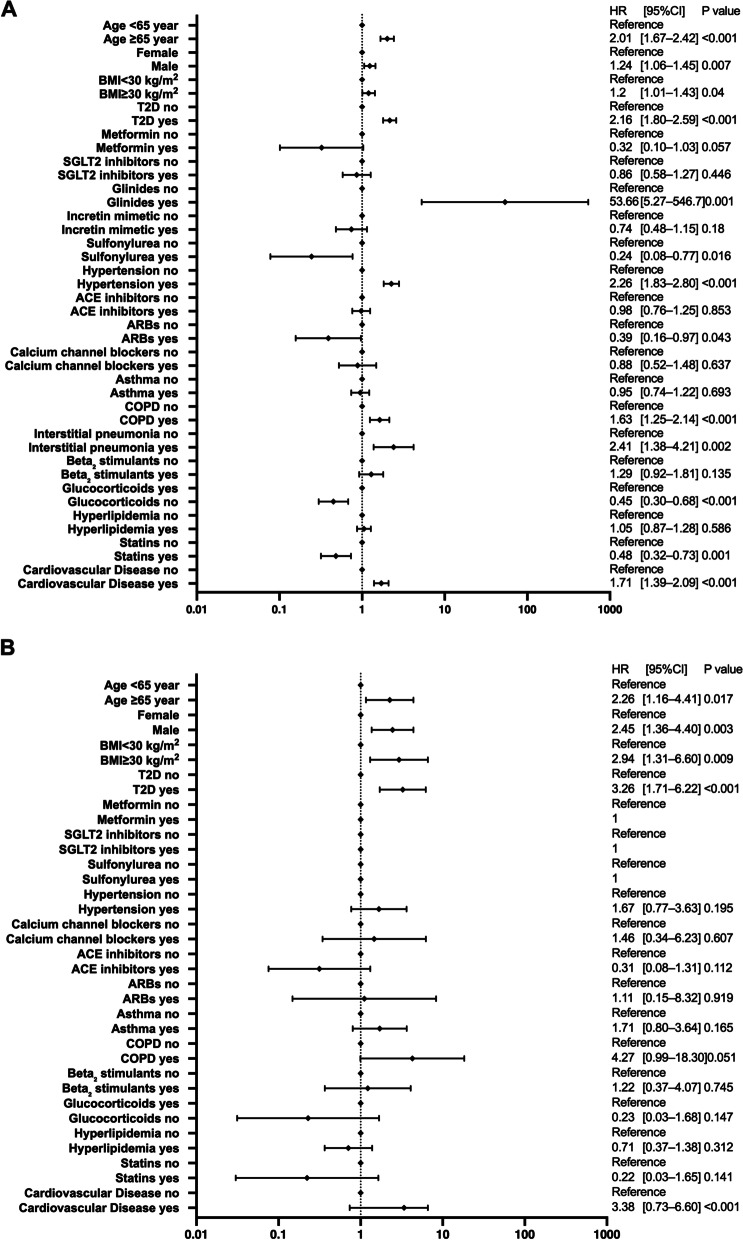
Analysis of hospitalization and critical-care factors in Hispanic COVID-19 patients. The forest plot shows the HRs (diamonds) and 95% CIs (horizontal bars) for hospitalization risk (**A**) and critical care (**B**). ACE, angiotensin-converting enzyme; ARB, angiotensin receptor blocker; BMI, body mass index; CI, confidence interval; COPD, chronic obstructive pulmonary disease; COVID-19, coronavirus disease 2019; HRs, hazard ratio; SGLT2, sodium-glucose cotransporter-2; T2D, type 2 diabetes

**Fig. 6:**
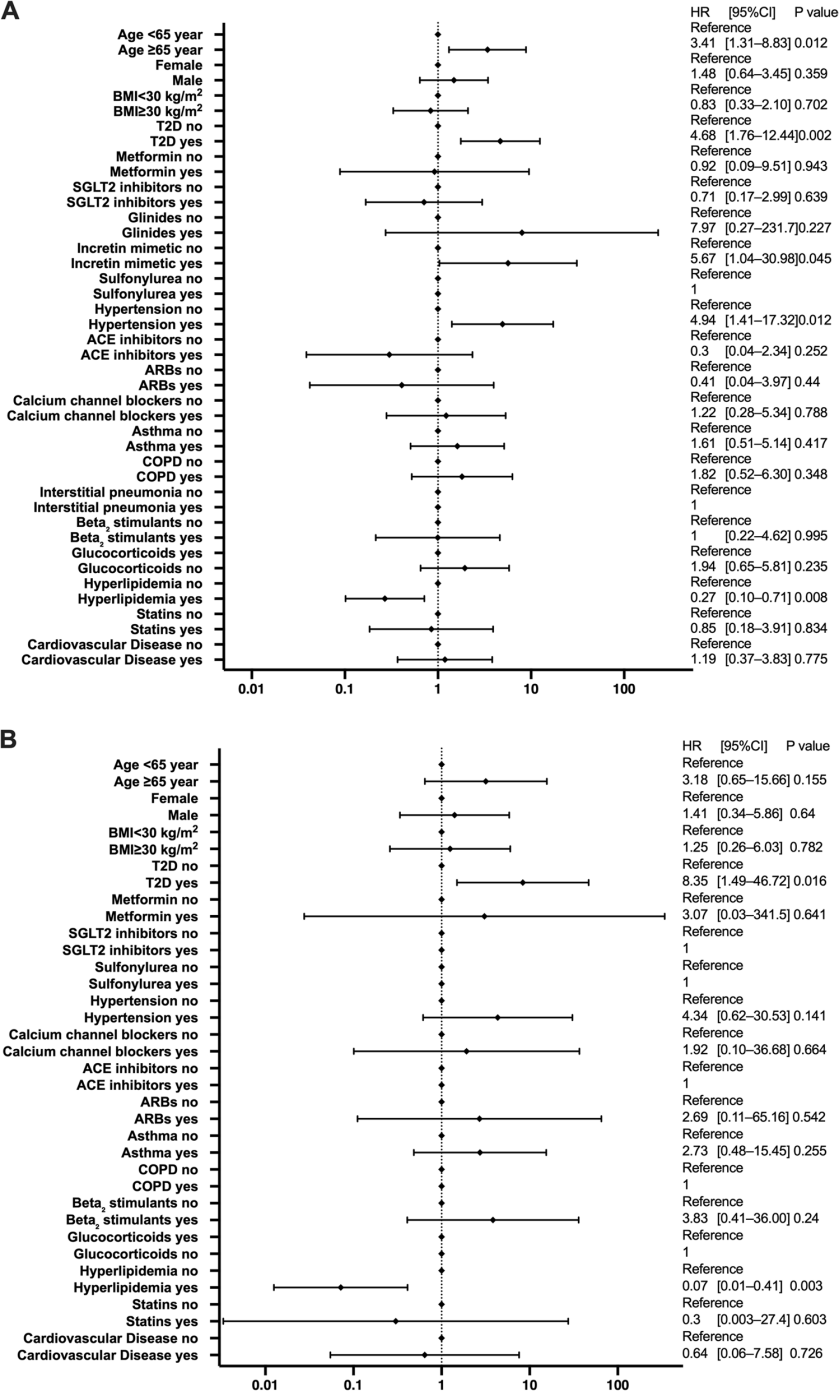
Analysis of hospitalization and critical-care factors in Asian COVID-19 patients. The forest plot shows the HRs (diamonds) and 95% CIs (horizontal bars) for hospitalization risk (**A**) and critical care (**B**). ACE, angiotensin-converting enzyme; ARB, angiotensin receptor blocker; BMI, body mass index; CI, confidence interval; COPD, chronic obstructive pulmonary disease; COVID-19, coronavirus disease 2019; HRs, hazard ratio; SGLT2, sodium-glucose cotransporter-2; T2D, type 2 diabetes

The results of the multivariate analysis by race are shown in Fig. 3 for non-Hispanic White patients, Fig. 4 for Black patients, Fig. 5 for Hispanic patients, and Fig. 6 for Asian patients.

Diabetes increased the risk of hospitalization and critical care significantly for all races. Hispanic and non-Hispanic Black men had a significantly increased risk of hospitalization and critical care. In contrast, ACE inhibitors significantly reduced both the risk of hospitalization and critical care in non-Hispanic White patients and non-Hispanic Black patients, and reduced the risk of hospitalization in Hispanic patients. SGLT2 inhibitors significantly reduced the risk of hospitalization and critical care in non-Hispanic Black patients. Age 65 years and older and having a history of cardiovascular diseases significantly increased the risk of hospitalization and critical care for all races except Asian patients.

### Differences in risk of hospitalization and critical care among regional groups

**Fig. 7:**
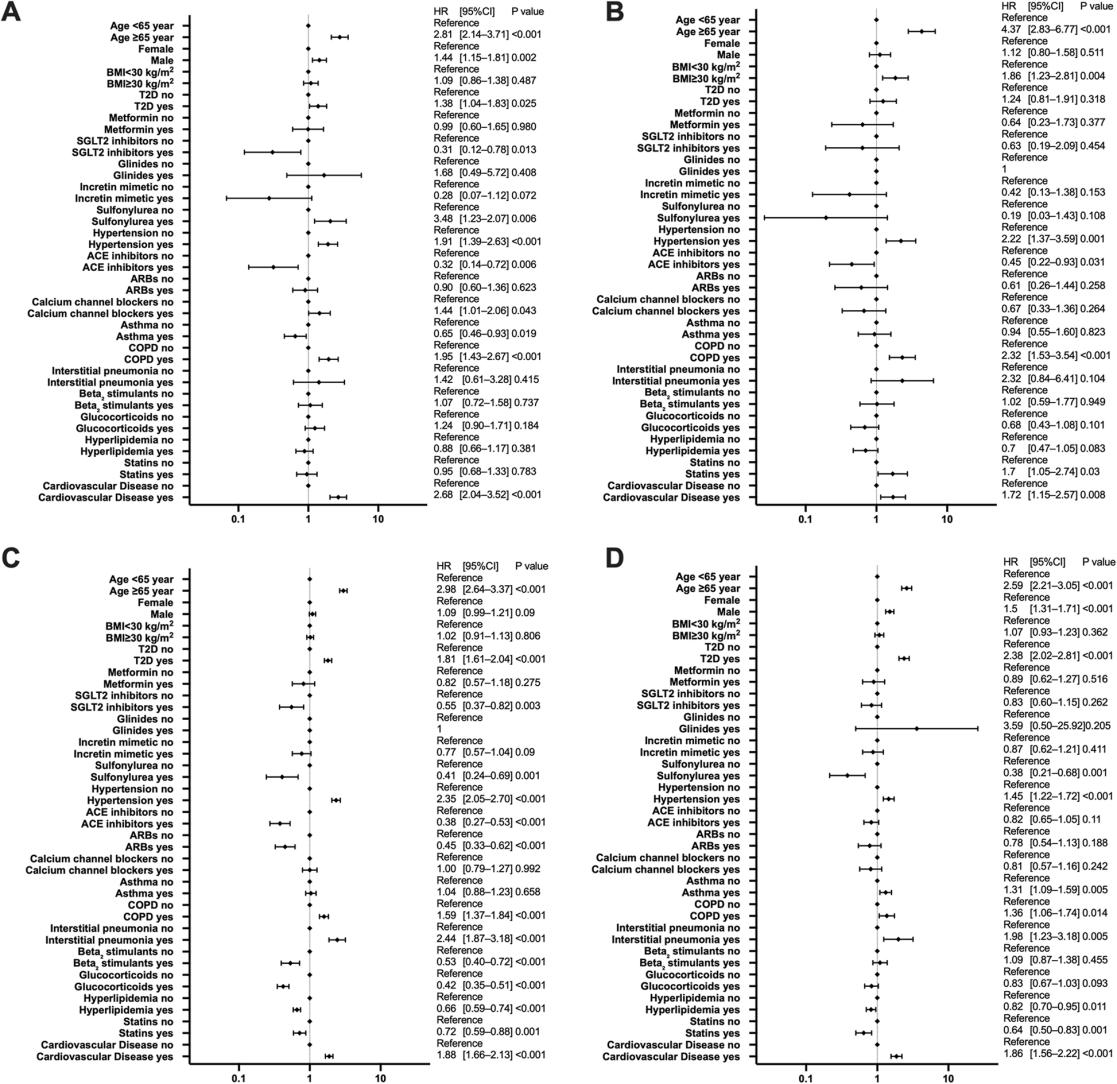
Analysis of hospitalization factors in COVID-19 patients in four regions of the United States. The forest plot shows the HRs (diamonds) and 95% CIs (horizontal bars) for the risk of hospitalization. Region of Northeast (**A**), Midwest (**B**), South (**C**), and West (**D**). ACE, angiotensin-converting enzyme; ARB, angiotensin receptor blocker; BMI, body mass index; CI, confidence interval; COPD, chronic obstructive pulmonary disease; COVID-19, coronavirus disease 2019; HRs, hazard ratio; SGLT2, sodium-glucose cotransporter-2; T2D, type 2 diabetes

**Fig. 8:**
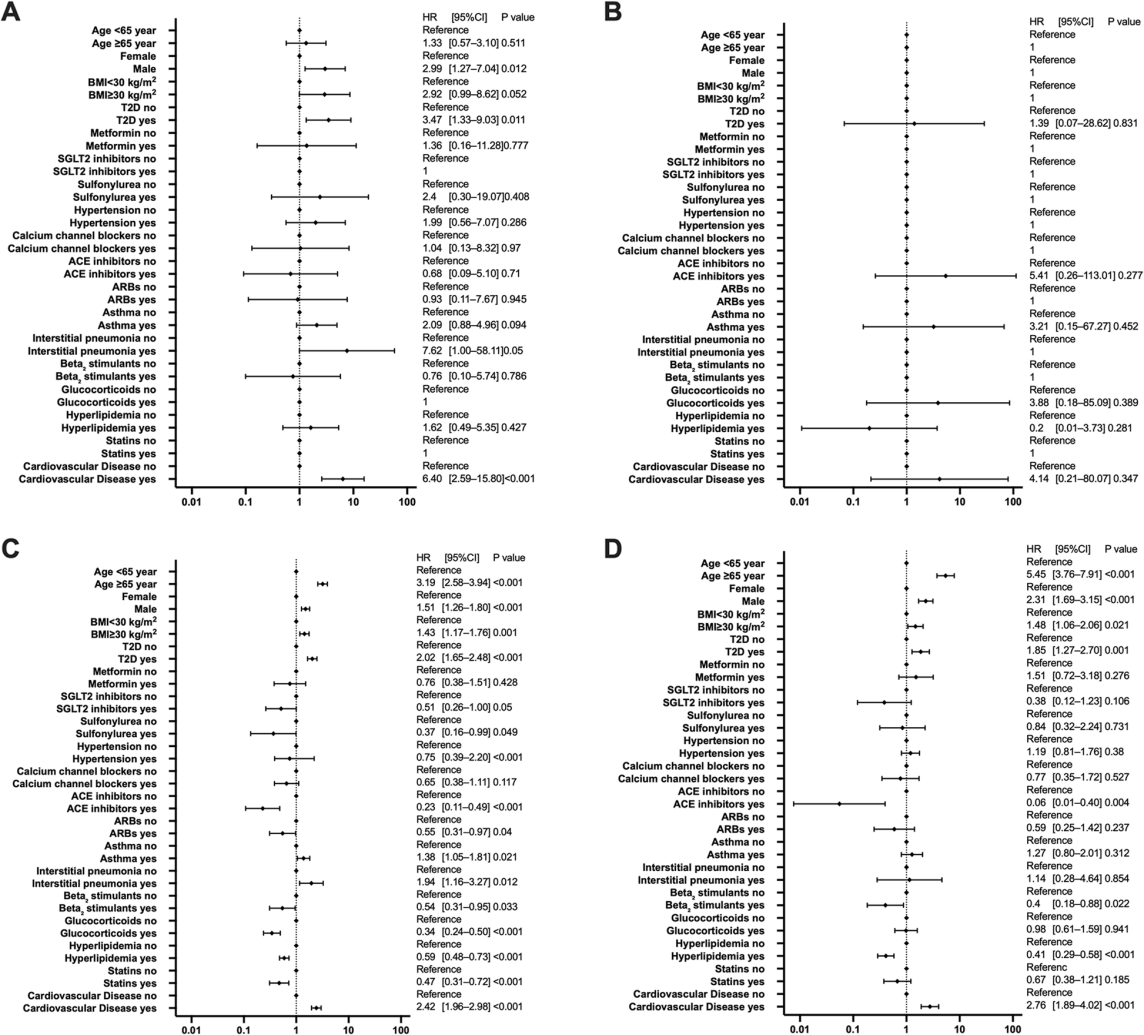
Sub-analysis of critical-care factors in COVID-19 patients by four regions of the United States. The forest plot shows HRs (diamonds) and 95% CIs (horizontal bars) for the risk of critical care. Region of Northeast (**A**), Midwest (**B**), South (**C**), and West (**D**). ACE, angiotensin-converting enzyme; ARB, angiotensin receptor blocker; BMI, body mass index; CI, confidence interval; COPD, chronic obstructive pulmonary disease; COVID-19, coronavirus disease 2019; HR, hazard ratio; SGLT2, sodium-glucose cotransporter-2; T2D, type 2 diabetes

The results of the multivariate analysis by region are presented in Figs. 7 and 8. The factors that significantly increased the risk of hospitalization in all regions were older age, hypertension, COPD, and cardiovascular disease. ACE inhibitors significantly reduced the risk of hospitalization in the Northeast, Midwest, and South and significantly reduced the risk of critical care in the South and West.

## Discussion

Consistent with previous results, this study showed that older age, male sex, and coexisting type 2 diabetes, hypertension, interstitial pneumonia, COPD, and cardiovascular disease were associated with worse COVID-19 outcomes in the United States. This study also showed that comorbid hyperlipidemia and a history of SGLT2 inhibitor, ACE inhibitor, ARB, glucocorticoid, and statin use were associated with a decreased risk of COVID-19 severity.

### Type 2 diabetes drugs

Among type 2 diabetes drugs, SGLT2 inhibitors significantly reduced the HR. SGLT2 inhibitors are expected to reduce the severity of COVID-19 because of their anti-inflammatory, cytokine-stimulating, and cardioprotective effects [14]. However, a randomized, double-blind, placebo-controlled trial exploring the effect of dapagliflozin in hospitalized COVID-19 patients with cardiovascular metabolic risk factors, reported no improvement in clinical recovery [15]. There have been case reports of SGLT2 inhibitor administration in COVID-19 patients presenting with normoglycemic ketoacidosis [16]. If COVID-19 is suspected, we believe that it is safe to follow the basics of the sick day rule for diabetes and stop taking SGLT2 inhibitors.

The HR for hospitalization risk was significantly higher for glinides, and significantly lower for incretin mimetic and sulfonylurea. The present patient data included more than 2,000 patients each who were prescribed metformin, incretin mimetic, SGLT2 inhibitors, and sulfonylurea; however, fewer than 60 patients each were prescribed alpha-glucosidase inhibitors, dipeptidyl peptidase 4(DPP4) inhibitors, and glinides. Differences in sample size may have affected the results. Khunti et al. reported that they found no association between patients using glinide preparations and COVID-19-related deaths [8]. To determine whether glinide use affects COVID-19 severity, studies with larger sample sizes are necessary. Incretin mimetic have been reported to have anti-inflammatory and cardioprotective effects [17] and counteract the downregulation of ACE2 due to diabetes [18]. In addition, incretin mimetic could have reduced the severity of COVID-19 because of their ability to suppress obesity [19].

In contrast, there are reports that sulfonylurea, which significantly lowered the HR for hospitalization, has no effects on mortality due to COVID-19 [20]. We believe that the association between sulfonylurea agents and the severity of COVID-19 is low because the cardiovascular disease-preventive effect of sulfonylurea agents has not been confirmed [21]. Metformin has been reported to reduce the risk of COVID-19 severity [22]; while the present results showed a trend toward reduced risk, no significant difference was observed. Some studies have indicated that the use of metformin in COVID-19 patients predominantly increased the incidence of acidosis [23, 24]. Further studies are needed to determine the safe use of metformin in patients with COVID-19.

### ACE inhibitors and ARBs

Among antihypertensive drugs, ACE inhibitors and ARBs significantly reduced the HR. Both of these agents are involved in the renin-angiotensin system and have mechanisms that inhibit the action of angiotensin II. These drugs have been reported to upregulate ACE2, the infection receptor for COVID-19 [25], and could have reduced the rapid decrease in ACE2 expression caused by infection and subsequent lung injury and heart damage.

### Respiratory disease drugs

Among the drugs used to treat respiratory diseases, steroids resulted in a significantly lower HR. Yochai et al. reported that inhaled steroids exert anti-inflammatory effects in the lungs and reduce ACE2 and transmembrane protease, serine 2 expression in bronchial epithelial cells [26]. This suggests that reduced ACE2 expression may prevent COVID-19. However, the present analysis included both oral and inhaled medications. Steroids, such as dexamethasone, have been shown to be effective against severe disease since the early stages of the COVID-19 outbreak. It is possible that steroids were used to treat COVID-19 before hospitalization, thereby reducing the number of patients who were hospitalized or in critical care.

Beta_2_ stimulants also significantly reduced the HR for critical care. Yamaya et al. reported that in cold coronavirus (HCoV-229E), β_2_-stimulants suppressed viral receptor expression in cells [27]. Further studies are needed to determine whether COVID-19 suppresses receptor expression, and if it has other actions that reduce disease severity.

### Statins

Excluding patients who were prescribed statins resulted in a significantly higher HR for hospitalization in patients with hyperlipidemia, but no significant difference in the HR for critical care. Previous studies have suggested that hyperlipidemia is associated with severe COVID-19 [28]. Patients with hyperlipidemia have increased systemic cholesterol content, which may increase the ability of the virus to penetrate and infect host cells [29]. Statins have been shown to increase ACE2 expression, similarly to ACE inhibitors and ARBs, and may reduce lung and heart damage by preventing excess the action of angiotensin II [30]. Statins have also been shown to decrease the amount of cholesterol in lipid rafts, limit viral penetration into host cells, reduce serum levels of interleukin-6 (which is involved in cytokine storms), and protect vascular endothelium from free radicals [31]. Based on the above, we considered hyperlipidemia as a risk factor for COVID-19 severity; however, statins are likely to have a mechanism to reduce COVID-19 severity, resulting in a lower HR for patients. Further evaluation of statin efficacy in randomized controlled trials is necessary.

### Relationship between region, race and risk factors

In elderly men, diabetes and cardiovascular disease tended to increase the HRs in both region-specific and race-specific analyses. In contrast, ACE inhibitors tended to lower the HR. These could be risk factors for severe disease or factors that reduce the risk of severe disease, regardless of the region of residence or race.

The analysis by region showed a large variation in the number of patients, and it was not possible to determine the characteristics of differences in the risk factors for severe disease in different regions. In the race-specific analysis, non-Hispanic Black and Hispanic patients showed different trends from the other groups.

Risks of hospitalization and critical care were highest among non-Hispanic Black patients. non-Hispanic Black patients have been reported to be at significantly higher risk of death from COVID-19 than non-Hispanic White patients or Asian patients [32], suggesting that non-Hispanic Black patients could be more susceptible to severe disease. This may be due to the social background of non-Hispanic Black patients in the United States. African Americans, on average, have lower college grades than Whites and are disproportionately employed in essential occupations such as food, cleaning, and transportation industries [33], suggesting that they had more opportunities to come into contact with people during the pandemic. In addition, low-income individuals tend to consume more calorie-dense foods and are at risk for lifestyle-related diseases [34], making them more likely to experience more severe symptoms if infected with COVID-19. Social factors may significantly contribute to variations in hospitalization and critical care risk. Furthermore, the absence of insurance coverage and limited access to healthcare services could significantly contribute to disparities in the outcomes.

In addition, a unique feature of the results for non-Hispanic Black patients was that the item that most significantly reduced the HR for hospitalization was a SGLT2 inhibitor. Other racial groups did not show significant differences in SGLT2 inhibitor use, with only non-Hispanic Black patients exhibiting significantly lower results. Clements et al. reported that Black individuals with diabetes have a higher incidence of chronic kidney disease (CKD) comorbidity [35]. Chronic kidney disease has been suggested to increase mortality in COVID-19 patients, and could be associated with renoprotective SGLT2 inhibitors that reduce the severity of COVID-19 [36].

Hypertension was detected as a COVID-19 severity factor in the overall multivariate analysis; however, when analyzed by race, a no significant association was found Hispanic and non-Hispanic Asian patients. No significant association was observed in non-Hispanic Asian patients due to the small population size. Hispanic individuals have a lower prevalence of hypertension in the United States than do White and Black individuals. Hispanic individuals have been reported to have lower mortality rates from cardiovascular diseases than that of White individuals, suggesting that Hispanic persons differ from other racial groups in their risk for cardiovascular diseases [37]. Therefore, hypertension was less strongly associated with COVID-19 severity. However, there are currently no reports of specific mechanisms linking hypertension, cardiovascular disease, and COVID-19 in Hispanic individuals. It is possible that because Hispanic persons make up a smaller percentage of the insured population and have poorer access to healthcare, many of these individuals have hypertension but remain undiagnosed [7]. It may be necessary to determine whether there is a difference in COVID-19 severity between the groups with and without the diagnosis of hypertension.

### Limitations

The present study has some limitations. First, the underlying diseases of the patients were extracted based on their EHRs and did not reflect the stage or progression of their disease. Second, only diagnosed infections were included in the analysis; therefore, the impact of reinfection was not considered. Non-symptomatic infections that were not included in the database were also excluded. Third, although it was necessary to investigate the impact of vaccination to improve the accuracy of the data, missing information prevented identification of patients who were not vaccinated. Furthermore, vaccinations are administered rapidly at venues and pharmacies, making it difficult to link the information in this database. Fourth, this study did not examine the effects of malignancy, cerebrovascular disease, CKD, human immunodeficiency virus infection, immunodeficiency, immunosuppressive drugs, pediatric disease, or pregnancy. Fifth, because drug information in this case was analyzed based on the drug prescription history, it was not possible to determine whether a drug was actually administered to the patient. Finally, we did not examine the impact of multiple risk factors for severe disease or factors that reduce the risk of severe disease. Additionally, the present analysis was not conducted separately for each specific drug or disease management status. Further studies are needed to determine which patients are more likely or less likely to develop severe disease.

## Conclusions

One significant contribution of our study is the examination of ethnicity and race as risk factors for COVID-19 severity. We provide new findings regarding disparities in hospitalization and ICU admission rates among racial and regional groups within the US population. However, it is essential to note that our analysis may not cover all possible configurations of ethnicity and race in the United States, and the results should be interpreted with caution. In a cohort analysis of 171,491 COVID-19-positive patients from a large United States electronic health record (EHR) database, we observed that several factors increased the risk of hospitalization and critical care. These factors included age ≥ 65 years, male sex, type 2 diabetes, hypertension, interstitial pneumonia, and cardiovascular disease. In contrast, we found that certain medications, such as SGLT2 inhibitors, ACE inhibitors, ARBs, glucocorticoids, and statins, significantly reduced the risk of hospitalization and critical care. Furthermore, our study revealed that type 2 diabetes increased the risk of hospitalization and critical care across all ethnicities. Additionally, age ≥ 65 years, hypertension, COPD, and cardiovascular disease significantly increased the risk of hospitalization in all regions of the United States. These findings underscore the importance of appropriate drug therapy and management of lifestyle-related diseases in reducing the severity of COVID-19.

## Data Availability

The data that support the findings of this study are available from the Healthjump database and the COVID-19 Research Database consortium; however, restrictions apply to the availability of these data, which were used under license for the current study. Therefore, these data are not publicly available. However, data are available from the authors upon reasonable request, and with permission from the Healthjump database and COVID-19 Research Database consortium.

## Abbreviations

COVID-19: Coronavirus disease 2019
SGLT2: Sodium-glucose cotransporter-2
ACE: Angiotensin-converting enzyme
ARBs: Angiotensin II receptor blockers
HER: Electronic health record
BMI: Body mass index
ICD-10: International Statistical Classification of Diseases and Related Health Problems, Tenth Revision
CPT: Current Procedural Terminology
HCPCS: Healthcare Common Procedure Coding System
HRs: Hazard ratios
DPP4: Dipeptidyl peptidase 4
CKD: Chronic kidney disease
CI: Confidence interval
COPD: Chronic obstructive pulmonary disease
T2D: Type 2 diabetes

## References

[1] Richardson S, Hirsch JS, Narasimhan M, Crawford JM, McGinn T, Davidson KW (2020). Presenting Characteristics, Comorbidities, and Outcomes Among 5700 Patients Hospitalized With COVID-19 in the New York City Area. JAMA.

[2] Cummings MJ, Baldwin MR, Abrams D, Jacobson SD, Meyer BJ, Balough EM (2020). Epidemiology, Clinical Course, and Outcomes of Critically Ill Adults With COVID-19 in New York City: A Prospective Cohort Study. Lancet.

[3] Bennett TD, Moffitt RA, Hajagos JG, Amor B, Anand A, Bissell MM (2021). Clinical Characterization and Prediction of Clinical Severity of SARS-CoV-2 Infection Among US Adults Using Data From the US National COVID Cohort Collaborative. JAMA Netw Open..

[4] Williamson EJ, Walker AJ, Bhaskaran K, Bacon S, Bates C, Morton CE (2020). Factors Associated With COVID-19-Related Death Using OpenSAFELY. Nature.

[5] Mathur R, Rentsch CT, Morton CE, Hulme WJ, Schultze A, MacKenna B (2021). Ethnic Differences in SARS-CoV-2 Infection and COVID-19-Related Hospitalisation, Intensive Care Unit Admission, and Death in 17 Million Adults in England: An Observational Cohort Study Using the OpenSAFELY Platform. Lancet.

[6] Nau C, Bruxvoort K, Navarro RA, Chevez SG, Hogan TA, Ironside KR (2021). COVID-19 Inequities Across Multiple Racial and Ethnic Groups: Results From an Integrated Health Care Organization. Ann Intern Med.

[7] Bureau USC. Decennial Census of Population and Housing /by Decade /2020 Census Decade 2020. Available from: https://www.census.gov. Accessed 15 Jan 2022.

[8] Khunti K, Knighton P, Zaccardi F, Bakhai C, Barron E, Holman N (2021). Prescription of Glucose-Lowering Therapies and Risk of COVID-19 Mortality in People With Type 2 Diabetes: A Nationwide Observational Study in England. Lancet Diabetes Endocrinol.

[9] Reynolds HR, Adhikari S, Pulgarin C, Troxel AB, Iturrate E, Johnson SB (2020). Renin-Angiotensin-Aldosterone System Inhibitors and Risk of Covid-19. N Engl J Med.

[10] Baral R, Tsampasian V, Debski M, Moran B, Garg P, Clark A (2021). Association Between Renin-Angiotensin-Aldosterone System Inhibitors and Clinical Outcomes in Patients With COVID-19: A Systematic Review and Meta-analysis. JAMA Netw Open..

[11] Zhang XJ, Qin JJ, Cheng X, Shen L, Zhao YC, Yuan Y (2020). In-Hospital Use of Statins Is Associated With a Reduced Risk of Mortality Among Individuals With COVID-19. Cell Metab.

[12] Ando W, Horii T, Uematsu T, Hanaki H, Atsuda K, Otori K (2021). Impact of overlapping risks of type 2 diabetes and obesity on coronavirus disease severity in the United States. Sci Rep.

[13] Ando W, Horii T, Jimbo M, Uematsu T, Atsuda K, Hanaki H, et al. Smoking cessation in the elderly as a sign of susceptibility to symptomatic COVID-19 reinfection in the United States. Front Public Health. 2022;10:985494. 10.3389/fpubh.2022.985494. 10.3389/fpubh.2022.985494 PMC9733529 36504971

[14] Cowie MR, Fisher M (2020). SGLT2 Inhibitors: Mechanisms of Cardiovascular Benefit Beyond Glycaemic Control. Nat Rev Cardiol.

[15] Kosiborod MN, Esterline R, Furtado RHM, Oscarsson J, Gasparyan SB, Koch GG (2021). Dapagliflozin in Patients With Cardiometabolic Risk Factors Hospitalised With COVID-19 (DARE-19): A Randomised, Double-Blind, Placebo-Controlled, Phase 3 Trial. Lancet Diabetes Endocrinol.

[16] Vitale RJ, Valtis YK, McDonnell ME, Palermo NE, Fisher NDL (2021). Euglycemic Diabetic Ketoacidosis With COVID-19 Infection in Patients With Type 2 Diabetes Taking SGLT2 Inhibitors. AACE Clin Case Rep.

[17] Gerstein HC, Colhoun HM, Dagenais GR, Diaz R, Lakshmanan M, Pais P (2019). Dulaglutide and Cardiovascular Outcomes in Type 2 Diabetes (REWIND): A Double-Blind. Randomised Placebo-Controlled Trial Lancet.

[18] Drucker DJ. Coronavirus Infections and Type 2 Diabetes-Shared Pathways With Therapeutic Implications. Endocr Rev. 2020;41:bnaa011. 10.1210/endrev/bnaa011. 10.1210/endrev/bnaa011 PMC7184382 32294179

[19] Perez-Montes DE, Oca A, Pellitero S, Puig-Domingo M (2021). Obesity and GLP-1. Minerva Endocrinol (Torino).

[20] Nguyen NN, Ho DS, Nguyen HS, Ho DKN, Li HY, Lin CY, et al. Preadmission Use of Antidiabetic Medications and Mortality Among Patients With COVID-19 Having Type 2 Diabetes: A Meta-analysis. Metabolism. 2022;131:155196. 10.1016/j.metabol.2022.155196. 10.1016/j.metabol.2022.155196 PMC8970613 35367460

[21] Rosenstock J, Kahn SE, Johansen OE, Zinman B, Espeland MA, Woerle HJ (2019). Effect of Linagliptin vs Glimepiride on Major Adverse Cardiovascular Outcomes in Patients With Type 2 Diabetes: The CAROLINA Randomized Clinical Trial. JAMA.

[22] Li Y, Yang X, Yan P, Sun T, Zeng Z, Li S. Metformin in Patients With COVID-19: A Systematic Review and Meta-analysis. Front Med (Lausanne). 2021;8:704666. 10.3389/fmed.2021.704666. 10.3389/fmed.2021.704666 PMC8416892 34490296

[23] Cheng X, Liu YM, Li H, Zhang X, Lei F, Qin JJ (2020). Metformin Is Associated With Higher Incidence of Acidosis, but Not Mortality, in Individuals With COVID-19 and Pre-existing Type 2 Diabetes. Cell Metab.

[24] Crouse AB, Grimes T, Li P, Might M, Ovalle F, Shalev A. Metformin Use Is Associated With Reduced Mortality in a Diverse Population With COVID-19 and Diabetes. Front Endocrinol. 2020;11:600439. 10.3389/fendo.2020.600439. 10.3389/fendo.2020.600439 PMC7838490 33519709

[25] Semenzato L, Botton J, Drouin J, Baricault B, Vabre C, Cuenot F (2021). Antihypertensive Drugs and COVID-19 Risk: A Cohort Study of 2 Million Hypertensive Patients. Hypertension.

[26] Adir Y, Saliba W, Beurnier A, Humbert M. Asthma and COVID-19: An Update. Eur Respir Rev. 2021;30. 10.1183/16000617.0152-2021. 10.1183/16000617.0152-2021 PMC8674937 34911694

[27] Yamaya M, Nishimura H, Deng X, Sugawara M, Watanabe O, Nomura K (2020). Inhibitory Effects of Glycopyrronium, Formoterol, and Budesonide on Coronavirus HCoV-229E Replication and Cytokine Production by Primary Cultures of Human Nasal and Tracheal Epithelial Cells. Respir Investig.

[28] Liu Y, Pan Y, Yin Y, Chen W, Li X (2021). Association of Dyslipidemia With the Severity and Mortality of Coronavirus Disease 2019 (COVID-19): A Meta-analysis. Virol J.

[29] Tang Y, Hu L, Liu Y, Zhou B, Qin X, Ye J (2021). Possible Mechanisms of Cholesterol Elevation Aggravating COVID-19. Int J Med Sci.

[30] Torres-Peña JD, Katsiki N, Perez-Martinez P. Could Statin Therapy Be Useful in Patients With Coronavirus Disease 2019 (COVID-19)? Front Cardiovasc Med. 2021;8:775749. 10.3389/fcvm.2021.775749. 10.3389/fcvm.2021.775749 PMC8578478 34778421

[31] Surma S, Banach M, Lewek J. COVID-19 and Lipids. The Role of Lipid Disorders and Statin Use in the Prognosis of Patients With SARS-CoV-2 Infection. Lipids Health Dis. 2021;20:141. 10.1186/s12944-021-01563-0. 10.1186/s12944-021-01563-0 PMC8542506 34689776

[32] Rushovich T, Boulicault M, Chen JT, Danielsen AC, Tarrant A, Richardson SS (2021). Sex Disparities in COVID-19 Mortality Vary Across US Racial Groups. J Gen Intern Med.

[33] Selden TM, Berdahl TA (2020). COVID-19 And Racial/Ethnic Disparities In Health Risk, Employment, And Household Composition. Health Aff (Millwood).

[34] Manyanga T, Tremblay MS, Chaput JP (2017). Socioeconomic status and dietary patterns in children from around the world: different associations by levels of country human development?. BMC Public Health.

[35] Clements JM, West BT, Yaker Z, Lauinger B, McCullers D, Haubert J, et al. Disparities in Diabetes-Related Multiple Chronic Conditions and Mortality: The Influence of Race. Diabetes Res Clin Pract. 2020;159:107984. 10.1016/j.diabres.2019.107984. 10.1016/j.diabres.2019.107984 PMC6959124 31846667

[36] Chung EYM, Palmer SC, Natale P, Krishnan A, Cooper TE, Saglimbene VM (2021). Incidence and Outcomes of COVID-19 in People With CKD: A Systematic Review and Meta-analysis. Am J Kidney Dis.

[37] Dominguez K, Penman-Aguilar A, Chang MH, Moonesinghe R, Castellanos T, Rodriguez-Lainz A (2015). Vital Signs: Leading Causes of Death, Prevalence of Diseases and Risk Factors, and Use of Health Services among Hispanics in the United States – 2009–2013 Vital Signs. MMWR Morb Mortal Wkly Rep.

